# 

**Published:** 1889-08

**Authors:** 


### CONTENTS:

Article I.—Care of Children. . . . . . . . 145
" II.—Etiology of Caries. By W. C. Wardlaw, M. D., D. D. S . . . . . . . 154
" III.—Dissemination of the knowledge of Dental Hygiene Among the Masses By L. P. Bethel., D D. S. . . . . . 163
" IV.—Separate the Operative from the Mechanical. By Dr. W. W. Allport. . . . . . 170
An Explanation. . . . . . . . . . . 175
The Southern Dental Association. . . . . . . . 181

### EDITORIAL.

The Colorado Dental Law. . . . . . . . . 182
The Texas Dental Law. . . . . . . . . 184

### MONTHLY SUMMARY.

Anæsthetics in Dental Practice. . . . . . . . 187

Cold Water in Facial Neuralgia. . . . . . . . 189

Faults of Copper Amalgam. . . . . . . . . 190

